# Early Ventricular Arrhythmias Correlate with Adverse Outcome in Takotsubo Syndrome: Analysis of a Large Single-Center Database

**DOI:** 10.3390/jcdd12110437

**Published:** 2025-11-06

**Authors:** Sati Güler-Eren, Fatih Güner, Charleen-Therese Wanjek, Hilke Könemann, Nawar Alhourani, Fabienne Kreimer, Julian Wolfes, Benjamin Rath, Christian Ellermann, Julia Köbe, Florian Reinke, Gerrit Frommeyer, Felix Wegner, Lars Eckardt

**Affiliations:** Department of Cardiology II: Electrophysiology, University Hospital Münster, Albert-Schweitzer Campus 1, 48149 Münster, Germany; fatih.guener@ukmuenster.de (F.G.); c-t.scholtes@gmx.de (C.-T.W.); hilke.koenemann@ukmuenster.de (H.K.); nawar.alhourani@ukmuenster.de (N.A.); fabienne.kreimer@ukmuenster.de (F.K.); julian.wolfes@ukmuenster.de (J.W.); christian.ellermann@ukmuenster.de (C.E.); julia.koebe@ukmuenster.de (J.K.); florian.reinke@ukmuenster.de (F.R.); gerrit.frommeyer@ukmuenster.de (G.F.); felix.wegner@ukmuenster.de (F.W.); lars.eckardt@ukmuenster.de (L.E.)

**Keywords:** Takotsubo, acute arrhythmia, ventricular arrhythmia, outcome

## Abstract

Background: Takotsubo syndrome (TTS) is an acute cardiac condition characterized by transient left ventricular dysfunction. Although generally considered reversible, early arrhythmias are a dreaded complication and their prognostic significance remains incompletely understood. Methods: In this study, 104 consecutive patients diagnosed with TTS (January 2007 to September 2024) were examined for the prognostic relevance of in-hospital arrhythmias during monitoring at the time of diagnosis. The median follow-up was 2.1 years. The primary combined endpoint included cardiac death, TTS recurrence, occurrence of arrhythmias, and rehospitalization for cardiac causes. Results: In-hospital arrhythmias occurred in 35.6% of the patients. Ventricular arrhythmias were significantly associated with an increased risk of adverse cardiac events (odds ratio 3.94, 95% confidence interval 1.22–12.69; *p* = 0.021). Reduced left ventricular ejection fraction and QTc prolongation, while frequently observed, were not independently associated with adverse outcomes when analyzed separately from arrhythmic events. Supraventricular arrhythmias exhibited a non-significant trend (*p* = 0.145). Conclusions: In a large registry of consecutive TTS patients, in-hospital ventricular arrhythmias at diagnosis were significantly associated with adverse outcomes, underscoring the importance of early rhythm monitoring.

## 1. Introduction

Takotsubo syndrome (TTS), first described in Japan in 1990 as a cardiac syndrome [1], is defined as an acute cardiac condition characterized by transient left ventricular dysfunction, predominantly affecting postmenopausal women [2,3]. The syndrome is described as “stress cardiomyopathy”, while both negative (“broken heart syndrome”) [4] and positive stress (“happy heart syndrome”) [5] seem to be of significance. It often presents with symptoms like acute coronary syndrome, such as sudden-onset chest pain and dyspnea, and may also follow intense physical stress [6]. Coronary angiography usually reveals unobstructed coronary arteries, distinguishing TTS from myocardial infarction [7].

The exact pathophysiology of TTS is incompletely understood [8]. Stress hormones seem to play an important role, as elevated levels of catecholamines, particularly adrenaline or noradrenaline, have been observed in patients during the acute phase [9]. Neuroimaging has indicated increased activity in brain regions comprising the emotional–autonomic control system prior to TTS episodes, highlighting a potential neurocardiac link [10,11]. Experimental and clinical data suggest that catecholamine-mediated myocardial stunning, microvascular dysfunction, and coronary spasm contribute to transient ventricular dysfunction [8,12]. Echocardiographic findings in the hyperacute phase of TTS have demonstrated reversible wall-motion abnormalities consistent with catecholamine-mediated myocardial toxicity [13], supporting the concept of TTS as a multifactorial neuro-cardiac disorder involving sympathetic hyperactivity, endothelial dysfunction, and altered myocardial energetics.

Despite the general consideration of TTS as a reversible condition with a favorable outcome, studies have reported serious complications during the acute in-hospital phase, including cardiogenic shock, life-threatening arrhythmias, and death [14]. Approximately 4–14% of TTS patients experience significant ventricular arrhythmias (VAs), often associated with QT_c_ prolongation [15,16], or supraventricular arrhythmias (SVT) [17]. Several studies have demonstrated increased in-hospital mortality among TTS patients presenting with life-threatening VAs in the early phase but no association with adverse long-term outcomes [18,19]. In contrast, data from international registries suggests that early arrhythmias may have prognostic relevance during follow-up [20,21]. The prognostic role of early arrhythmias, particularly ventricular arrhythmias, in long-term TTS outcomes remains uncertain. Thus, the present study aimed to address this.

## 2. Methods

Between January 2007 and September 2024, we retrospectively identified 104 consecutive patients diagnosed with TTS from the electronic database of our clinic. Diagnosis was based on the established Mayo Clinic criteria [22]: (1) transient regional wall motion abnormalities of the left ventricle involving mid-ventricular segments, with or without apical involvement, and not limited to a single coronary artery territory; (2) no evidence of obstructive coronary artery disease or acute plaque rupture; (3) new electrocardiographic changes mimicking acute coronary syndrome or mild elevation of cardiac biomarkers; and (4) absence of alternative explanations such as pheochromocytoma or myocarditis.

The study was conducted in accordance with the ethical standards of the Declaration of Helsinki. The research protocol received approval from the local ethical committee. We collected and analyzed clinical data including patient demographics, cardiovascular risk factors, electro- and echocardiographic parameters, laboratory markers, and angiographic results. Electrocardiograms and echocardiograms were reviewed independently by at least two physicians, including one board-certified cardiologist.

Patients were stratified into two groups based on the presence or absence of arrhythmias during index hospitalization. Arrhythmias were classified as SVT, VAs, and bradyarrhythmias, as identified through ECG and continuous rhythm monitoring of ≥48 h. VAs were defined as ventricular fibrillation (VF), sustained ventricular tachycardia (VT; duration ≥ 30 s or requiring immediate intervention such as cardioversion or defibrillation), non-sustained VT (≥3 PVCs, duration < 30 s, self-terminating), and premature ventricular contractions (PVCs). PVCs were considered clinically relevant only when frequent (≥5000 PVCs per 24 h) or associated with symptoms. Isolated, asymptomatic PVCs were not classified as arrhythmic events. Telemetry monitoring was evaluated daily by a physician to assess the burden of PVCs. Bradyarrhythmias included sinus bradycardia (heart rate < 50 bpm accompanied by symptoms or hemodynamic instability), sinus arrest or pauses ≥ 3 s, and second-degree type II or third-degree AV block.

Follow-up duration was set at a minimum of two years for all patients. This time frame was chosen to ensure that the prognostic analyses would reflect long-term outcomes by minimizing the confounding influence of (1) the initial left ventricular dysfunction and (2) other diseases not related to the initial TTS, as left ventricular function typically normalizes within weeks to months after the acute event [3].

Major cardiac outcomes included cardiac death, rehospitalization for cardiac causes, recurrence of TTS, and new or recurrent arrhythmias. Additional outcomes included all-cause and non-cardiac death, and thromboembolic events. The combined primary endpoint was the occurrence of any major cardiac event during follow-up. All components of the composite endpoint were equally weighted. Non-cardiac deaths were recorded separately and excluded from the composite cardiac outcome. Covariates considered in the analysis included age at diagnosis, left ventricular ejection fraction (LVEF), left atrial size, QT_c_ interval, and standard cardiovascular risk factors such as hypertension, diabetes, dyslipidemia, obesity, and smoking status. A subgroup analysis was performed for patients with TTS occurring in the context of a life-threatening medical condition (e.g., respiratory failure, cardiogenic shock, sepsis).

### Statistics

Continuous variables were reported as means ± standard deviation (SD) for normally distributed data. Categorical variables were presented as absolute frequencies and percentages. Distribution of continuous variables was assessed using the Kolmogorov–Smirnov test. Comparisons between groups were performed using the independent samples *t*-test for normally distributed continuous variables and the Mann–Whitney U test for continuous variables. For categorical data, the Chi-square (χ^2^) test or Fisher’s exact test was applied. Binary logistic regression analysis was conducted to determine the association between clinical variables and the occurrence of adverse cardiac outcomes. Variables with a *p*-value ≤ 0.10 in univariate analysis were considered for inclusion in the multivariate regression model. Odds ratios (OR) with 95% confidence intervals (CI) were calculated for each covariate. Additionally, subgroup analysis was performed to assess the prognostic impact of different arrhythmia subtypes (supraventricular, ventricular, bradyarrhythmia), using dummy coding for multinomial logistic regression models. To further explore potential heterogeneity, patients were stratified into sustained and non-sustained VAs. Separate logistic regression models were applied to each subtype to evaluate associations with the composite cardiac outcome.

Pearson correlation was used to assess relationships between QT_c_ interval, LV function, LA size, and clinical outcomes.

All statistical tests were two-sided, and a *p*-value of <0.05 was considered statistically significant. Statistical analysis was conducted using IBM SPSS Statistics version 29.0.2.0 (IBM Corp., Armonk, NY, USA).

## 3. Results

### 3.1. Baseline Characteristics

Clinical data from 104 consecutive in-hospital patients diagnosed with Takotsubo syndrome (TTS) with a mean follow-up duration of 2.1 ± 0.25 years were analyzed (Table 1). The cohort was stratified into two groups based on the presence *(n* = 37, 35.6%) or absence (*n* = 67, 64.4%) of arrhythmias during the early in-hospital phase after diagnosis. The two groups were comparable in age, sex, and prevalence of cardiovascular risk factors. Patients with arrhythmias exhibited significantly longer QT_c_ intervals (468 ± 32 ms vs. 452 ± 29 ms; *p* = 0.018) and a higher prevalence of left ventricular dysfunction (78.4% vs. 43.3%; *p* = 0.003) with no significant differences in QRS duration or other baseline variables.

### 3.2. In-Hospital Arrhythmias

**Figure 1:**
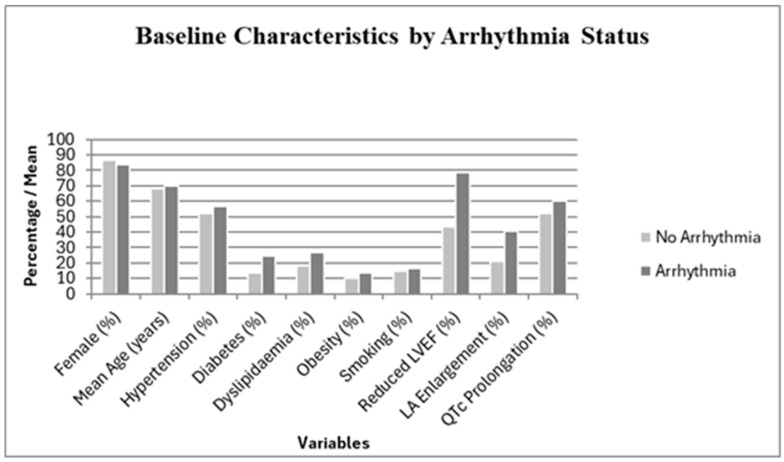
Comparison of patients with TTS with and without in-hospital arrhythmias in the early phase after diagnosis.

Among patients with arrhythmias, SVTs were observed in 14 individuals (13.5%), VAs—including non-sustained and sustained VAs or ventricular fibrillation (VF)—in 16 patients (15.4%), and bradyarrhythmias in 7 patients (6.7%). VF occurred in six patients, sustained ventricular tachycardia (VT) in three, and non-sustained VT or PVCs in seven patients. Notably, 31.3% of patients with VAs exhibited a prolonged QT_c_ interval exceeding 450 ms (Figure 1). Five patients died during their index hospitalization—two due to VAs, and the remainder from pulseless electrical activity, pneumonia with septic course and cardiogenic shock. The remaining 14 patients were discharged after a mean of 21.4 ± 6 days. Twelve of the VA patients (75%) received an implantable cardioverter defibrillator (ICD). Five patients (71.4%) were treated with a permanent pacemaker due to bradyarrhythmias. Two patients who experienced VAs followed by symptomatic bradyarrhythmias received a dual-chamber ICD (DDD-ICD).

### 3.3. Arrhythmias and Outcome

During follow-up, major clinical cardiac outcomes occurred: cardiac death (2.9%), rehospitalization for cardiac causes (33.7%), recurrence of TTS (1.9%), and new or recurrent arrhythmias (9.6%). These adverse cardiac events were significantly more frequent in patients with arrhythmias.

**Figure 2:**
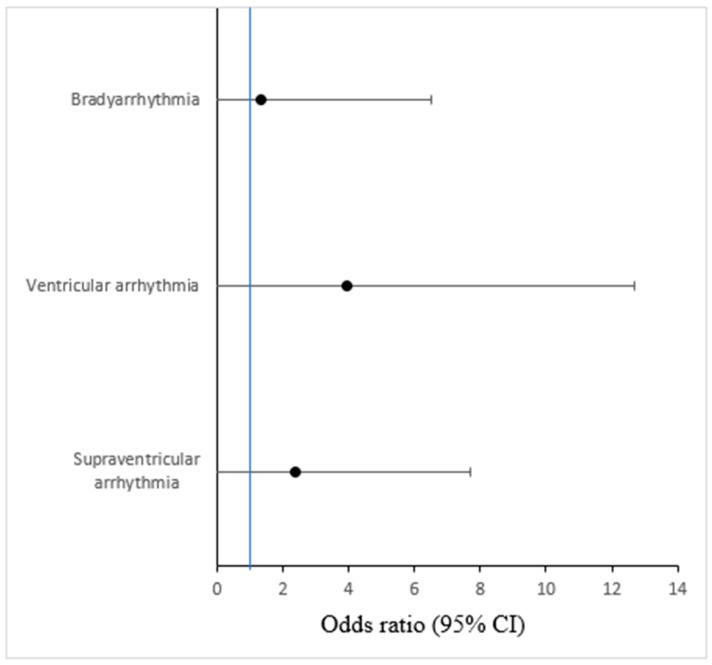
Association of arrhythmias with outcomes (cardiac death, TTS recurrence, occurrence of arrhythmias, and rehospitalization for cardiac cause). The blue vertical line indicates the reference line at OR = 1.

Binary logistic regression identified the presence of VAs during the initial in-hospital monitoring as a strong predictor of adverse outcomes (OR 3.94, 95% CI: 1.22–12.69; *p* = 0.021). In contrast, SVT demonstrated only a non-significant trend towards increased risk (OR 2.39; *p* = 0.145), and bradyarrhythmias showed no prognostic association (Figure 2).

In multivariate logistic regression, non-sustained VT was associated with an odds ratio of 3.79 (95% CI 0.70–20.61; *p* = 0.124), and sustained VT with an odds ratio of 3.03 (95% CI 0.71–12.91, *p* = 0.134).

### 3.4. Electrocardiographic Parameters and Cardiovascular Risk Factors

Although QT_c_ prolongation correlated with the presence of arrhythmias (*p* = 0.018), it was not independently associated with worse long-term outcome (r = 0.078, *p* = 0.432). Cardiovascular risk factors such as diabetes (*p* = 0.296), smoking (*p* = 0.75), dyslipidemia (*p* = 0.169), and obesity (*p* = 0.466) were also not associated with adverse events. Arterial hypertension showed a significant relationship with cardiac outcomes (*p* = 0.022).

Pearson correlation analysis demonstrated no significant correlation between QT_c_ and LV function (r = 0.137, *p* = 0.168), nor between LV function and clinical outcomes. However, the presence of arrhythmias and reduced LV function correlated significantly (*p* = 0.003) with clinical outcomes.

### 3.5. Subgroup Analysis of TTS Patients Triggered by Life-Threatening Medical Conditions

Patients with TTS precipitated by a life-threatening medical condition showed a non-significant trend towards a higher rate of adverse cardiac events (*p* = 0.066, Fisher’s exact test *p* = 0.107) (Table 2). When testing for an interaction between VAs and medical trigger status, no significant effect was observed (*p* = 0.999).

## 4. Discussion

In this retrospective study, we investigated the incidence and long-term prognostic role of arrhythmias in a cohort of 104 consecutive patients with Takotsubo syndrome (TTS). The main findings are the following: (1) Arrhythmias occurred in 35.6% of TTS patients, with VAs representing a strong predictor of adverse outcome during follow-up. In contrast, most of the classical risk factors such as LV function, diabetes, dyslipidemia, obesity and smoking were not significantly associated with outcome, except for arterial hypertension. (2) SVT and bradyarrhythmias did not show an association with long-term prognosis. (3) QT_c_ prolongation correlated with arrhythmias and outcomes in univariate analyses but did not persist as an independent risk factor in multivariable models.

Regarding the prevalence of arrhythmias in TTS patients, the large InterTAK Registry reported an overall arrhythmia rate of 9%, with VAs in only 3% of the cases [3]. Our cohort showed a higher prevalence of arrhythmias, particularly VAs. These differences may be attributed to the intensity of rhythm monitoring. Consistent with our findings, data from the InterTAK registry revealed a strong association between the occurrence of cardiac arrest and increased short- and long-term mortality in TTS patients [21]. Of note, left ventricular function was not an independent predictor of outcome in our cohort, suggesting that electrophysiologic instability is a stronger long-term risk factor than mechanical dysfunction. The association between VAs and outcome remained unchanged after accounting for medical triggers, and no interaction was observed. Thus, VAs represent a prognostic marker independent of the underlying precipitating condition.

The multicenter GErman Italian STress cardiomyopathy (GEIST) registry, which analyzed potentially life-threatening arrhythmias, including both VAs and bradyarrhythmias collectively [20], also observed a significantly higher short- and long-term mortality in TTS patients with arrhythmias in the acute in-hospital phase. However, in a retrospective study with a similar cohort size and demographic profile as compared to our study, El-Battrawy et al. [18] observed that life-threatening arrhythmias were primarily associated with cardiogenic shock, mechanical support, and increased in-hospital mortality with no significant differences in long-term outcomes between patients with and without ventricular arrhythmias. Our study examined a consecutive, unselected cohort using standardized diagnostic criteria and detailed rhythm monitoring, allowing assessment of arrhythmia subtypes and outcome. In contrast, InterTAK and GEIST predominantly analyzed registry-based populations with heterogeneous inclusion criteria. Differences in endpoint composition—e.g., our inclusion of non-fatal cardiac events in the composite outcome—may also account for the observed differences over time.

Importantly, our analysis evaluated VAs and bradyarrhythmias separately and additionally included non-sustained VT and PVCs. This approach revealed that the prognostic impact varies considerably between subtypes, with sustained VAs demonstrating a more consistent association with adverse long-term outcomes. Previous studies have indicated that patients with VAs may not benefit from ICDs whereas patients with bradyarrhythmias should undergo pacemaker implantation [23,24]. Our data indicate that early VAs are clinically relevant, but whether they warrant prophylactic or long-term device-based intervention cannot be concluded without prospective evidence. Thus, patients presenting with VAs should be prioritized for prolonged monitoring and structured follow-up, while other strategies should be guided by future prospective data rather than current retrospective trends.

The link between VAs and adverse long-term outcomes may reflect underlying structural or functional abnormalities, which are not completely reversible after acute TTS. Persistent myocardial inflammation, fibrosis, or autonomic imbalance have been described as possible substrates for both arrhythmogenesis and future cardiac events [8]. Previous studies have also shown that QT_c_ prolongation is a potential risk factor for intra-hospital complications [25,26]. Our study also shows a correlation between QT_c_ duration and arrhythmias, but a significant link between QT_c_ and all-cause cardiac events or mortality was not found, suggesting that QT_c_ is a sensitive but not specific risk marker requiring contextual interpretation.

### Study Limitations

Several limitations of our study should be acknowledged. The retrospective, single-center design limits the generalizability of our findings. The results on the prognostic relevance of early VAs in TTS should also be interpreted with caution due to the small sample size, which, although comparable to other single-center studies, resulted in limited statistical power and wide confidence intervals. The sample size further restricts the statistical power for subgroup analyses; for example, in TTS patients with a medical condition as an identified trigger, no reliable estimate of the coefficient was possible because of the small sample size and events within the combined subgroups.

Arrhythmia detection relied on ECG and in-hospital telemetry, which may underestimate transient, asymptomatic or post-discharge arrhythmias. Cause-specific mortality was partly dependent on available medical records and may be subject to misclassification bias. The high proportion of patients with VAs who received an ICD (75%) may have influenced long-term outcome by preventing arrhythmic deaths, potentially attenuating survival differences between subgroups. Finally, while the minimum follow-up duration was set at two years, changes in patient comorbidities and treatment strategies over time could have influenced the outcomes. Prospective multicenter studies with standardized arrhythmia monitoring protocols are needed to validate our findings.

## 5. Conclusions

In a large single-center registry of consecutive patients, early VAs emerged as the strongest predictor of adverse outcome in TTS and should be considered alongside hemodynamic markers such as left ventricular function and QT_c_ duration. The results highlight the potential prognostic relevance of arrhythmia phenotype and support the need for structured monitoring and individualized risk assessment in TTS. They provide a basis for future prospective studies evaluating prolonged monitoring and targeted therapeutic strategies.

## Figures and Tables

**Table 1:** Baseline characteristics stratified by arrhythmia status.

Variable	Arrhythmias (*n* = 37)	No Arrhythmias (*n* = 67)	*p*-Value
Female sex (%) Age at diagnosis (years)	31 (83.8%) 69.7 ± 10.2	58 (86.6%) 67.9 ± 11.8	0.724 0.361
QRS duration (ms)	96.5 ± 12.4	92.1 ± 10.3	0.082
QT_c_ interval (ms)	468.2 ± 32.5	451.7 ± 28.9	0.018 *
LV dysfunction at diagnosis (%)	29 (78.4%)	29 (43.3%)	0.003 *
Hypertension (%)	21 (56.8%)	35 (52.2%)	0.642
Diabetes mellitus (%)	9 (24.3%)	9 (13.4%)	0.192
Dyslipidemia (%)	10 (27.0%)	12 (17.9%)	0.177
Smoking (%)	6 (16.2%)	10 (14.9%)	0.698
Obesity (%)	5 (13.5%)	7 (10.4%)	0.650

Baseline clinical characteristics of TTS patients stratified by arrhythmia status. Patients with arrhythmias presented with significantly longer QT_c_ intervals and a higher prevalence of left ventricular dysfunction at diagnosis compared to patients without arrhythmias. * *p* indicates statistical significance.

**Table 2:** Baseline characteristics and outcomes (cardiac death, TTS recurrence, occurrence of arrhythmias, and rehospitalization for cardiac causes) according to trigger type.

Variable	Non-Medical Trigger (*n* = 93)	Medical Trigger (*n* = 11)	*p*-Value
Age at diagnosis (years)	66.4 ± 14.2	64.7 ± 12.0	0.64
Female sex, *n* (%)	80 (86.0%)	8 (72.7%)	0.28
LV dysfunction at diagnosis, *n* (%)	71 (76.3%)	10 (90.9%)	0.34
Composite adverse outcome, *n* (%)	44 (47.3%)	2 (18.2%)	0.066

Patients with life-threatening medical triggers showed similar age, sex distribution, and left-ventricular systolic function compared with those with non-medical triggers.

## Data Availability

The datasets generated and/or analyzed during the current study contain sensitive patient information and are therefore not publicly available due to ethical and legal restrictions (in accordance with the General Data Protection Regulation). The data will be shared on reasonable request to the corresponding author.
